# Deep learning for automatic Gleason pattern classification for grade group determination of prostate biopsies

**DOI:** 10.1007/s00428-019-02577-x

**Published:** 2019-05-16

**Authors:** Marit Lucas, Ilaria Jansen, C. Dilara Savci-Heijink, Sybren L. Meijer, Onno J. de Boer, Ton G. van Leeuwen, Daniel M. de Bruin, Henk A. Marquering

**Affiliations:** 1 Department of Biomedical Engineering and Physics, Amsterdam UMC, University of Amsterdam, Amsterdam, The Netherlands; 2 Department of Urology, Amsterdam UMC, University of Amsterdam, Amsterdam, The Netherlands; 3 Department of Pathology, Amsterdam UMC, University of Amsterdam, Amsterdam, The Netherlands; 4 Department of Radiology and Nuclear Medicine, Amsterdam UMC, University of Amsterdam, Amsterdam, The Netherlands

**Keywords:** Prostate, Gleason patterns, Grade groups, Convolutional neural network

## Abstract

Histopathologic grading of prostate cancer using Gleason patterns (GPs) is subject to a large inter-observer variability, which may result in suboptimal treatment of patients. With the introduction of digitization and whole-slide images of prostate biopsies, computer-aided grading becomes feasible. Computer-aided grading has the potential to improve histopathological grading and treatment selection for prostate cancer. Automated detection of GPs and determination of the grade groups (GG) using a convolutional neural network. In total, 96 prostate biopsies from 38 patients are annotated on pixel-level. Automated detection of GP 3 and GP ≥ 4 in digitized prostate biopsies is performed by re-training the Inception-v3 convolutional neural network (CNN). The outcome of the CNN is subsequently converted into probability maps of GP ≥ 3 and GP ≥ 4, and the GG of the whole biopsy is obtained according to these probability maps. Differentiation between non-atypical and malignant (GP ≥ 3) areas resulted in an accuracy of 92% with a sensitivity and specificity of 90 and 93%, respectively. The differentiation between GP ≥ 4 and GP ≤ 3 was accurate for 90%, with a sensitivity and specificity of 77 and 94%, respectively*.* Concordance of our automated GG determination method with a genitourinary pathologist was obtained in 65% (*κ* = 0.70), indicating substantial agreement. A CNN allows for accurate differentiation between non-atypical and malignant areas as defined by GPs, leading to a substantial agreement with the pathologist in defining the GG.

**Electronic supplementary material:**

The online version of this article (10.1007/s00428-019-02577-x) contains supplementary material, which is available to authorized users.

## Introduction

Prostate cancer is the second-most diagnosed cancer among men, accounting for approximately 25% of cancer cases in the western world [1]. It has been suggested that these high incidence rates are caused by widespread prostate-specific antigen (PSA) screening and subsequent biopsy harvesting [2].

Pathological grading of prostate cancer is originally based on the sum of the two most common Gleason patterns (GPs), called the Gleason score (GS) [3]. The initial Gleason grading system defines five histological patterns, with a focus on atypical glandular structures. GP 1 represents well-differentiated carcinoma, whereas GP 5 is defined as the least-differentiated carcinoma with complete loss of glandular structures. The intermediate scores are based on a linear scaling between the two extremes. Updates of the ISUP guidelines discouraged the assignment of Gleason scores 2–4. This was due to the poor reproducibility, poor correlation with radical prostatectomy grade, and deception of clinicians and patients, believing that there was an indolent tumor [4]. Better correlation with clinical outcome was achieved by the introduction of the modified Gleason score (GS) where the most frequently found GP and the highest GP are summed up.

The recently proposed grade groups (GGs) [5, 6] are aimed to more accurately predict the prognosis of patients. Even though the GG classification results in prognostic distinct grade groups [7], similar inter-observer variability rates to conventional Gleason scoring have been reported [8, 9]. Therefore, to avoid suboptimal treatments [5, 10, 11], an accurate and reproducible method to stratify the tumors is needed.

With the introduction of whole slide image (WSI) scanners, the digitization of slides has opened up the opportunity for computer-aided diagnosis (CAD), which has the potential to aid the pathologist and reduce inter-observer variability [12, 13]. Several studies have presented automated differentiation of GPs [12, 14–16]. Convolutional neural networks (CNNs), a deep learning approach particularly useful for the classification of images, nowadays allow the computer to automatically find the best set of image-based features. These features are able to distinguish between the predefined classes [17] without the dependency on extensive pre-processing or human knowledge. Litjens et al. [18] were able to automatically differentiate between tumorous and non-tumorous prostate biopsies using a CNN. Ing et al. [19] used semantic segmentation for the grading whole mount radical prostatectomy sections. Källén et al. [20] differentiated between GP 3 and GP 5 yielding an accuracy of 81% in homogeneous GP regions of interest within a biopsy. In this study, we propose an approach in which we include the extent of GP 3 and GP 4 patterns in heterogeneous biopsies for a whole slide GG classification.

## Methods

### Patient selection

The Institutional Review Board of the Amsterdam University Medical Centers (UMC), location AMC, Amsterdam (W18_056 # 18.074) granted approval for this study. Hematoxylin and eosin (H&E) tissue sections were retrieved from the archives of the department of Pathology of the Amsterdam UMC, location AMC. The sections originated from patients that underwent a diagnostic biopsy between 2015 and 2017 (*n* = 38). The H&E-stained 4-μm-thick sections were digitized using a Philips UltraFast scanner (Philips Digital Pathology Solutions, Best, the Netherlands) and the WSIs were exported at 20× magnification, resulting in a pixel resolution of 0.5 μm. A total of 96 tissue sections were included, which can contain multiple biopsies or biopsy fragments, derived from 38 patients, with a median of two tissue blocks per patient and an interquartile range of 1 to 4.

### Reference standard: manual annotations

**Fig. 1:**
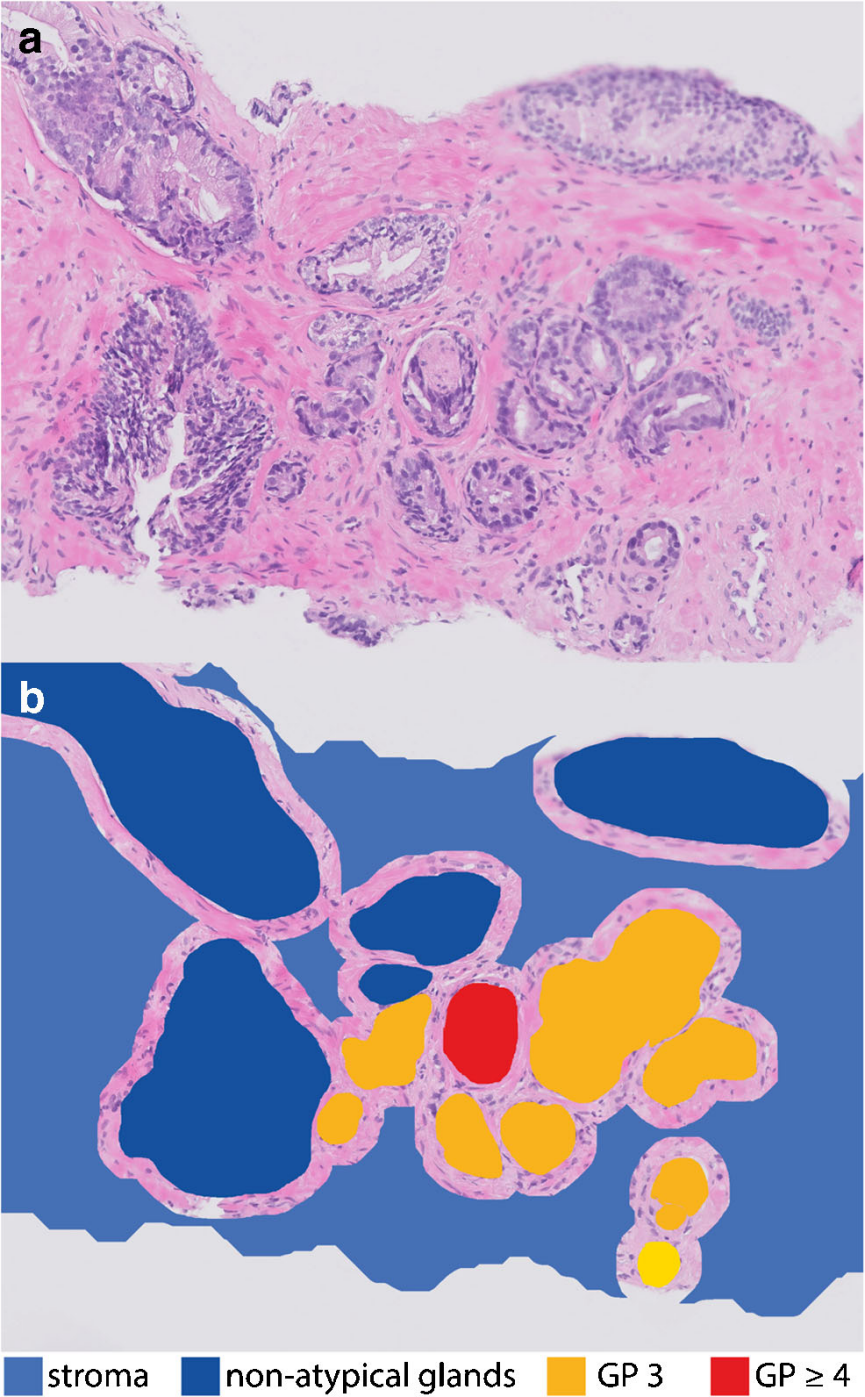
**a** H&E image **b** with the annotations of the four classes

The digitized slides were manually annotated by one of the two expert observers (I.J., KK.d.L.) and subsequently checked by a genitourinary pathologist (CD.S-H.) using an in-house developed free-hand annotation tool [21] (see Fig. 1). The first class was the unaffected stroma (connective tissue) of the prostate and was assigned to all pixels that were not in the proximity of other annotations. The non-atypical glands, including both healthy glands and glands with low-grade prostatic intraepithelial neoplasia (LGPIN), were defined as the second class. The third class was GP 3 and the fourth class consisted of GP ≥ 4 with the affected stroma. As the incidence of GP 5 was very low in this dataset, the GP 4 and GP 5 were merged to balance the classes. Subsequently, the GG for each biopsy was determined based on the surface area of GP 3 and GP ≥ 4 for each biopsy. As no differentiation was made between GP 4 and GP 5, a slightly adjusted grouping was applied, see Table 1.

**Table 1:** Graded groups and adjusted grade groups classification with the corresponding Gleason score

Grade groups	Gleason score	Adjusted grade groups	Gleason score
1	≤ 6	1	≤ 6
2	3 + 4	2	3 + ≥ 4
3	4 + 3	3	≥ 4 + 3
4	8	4	≥ 4 + ≥ 4
5	9–10		

When it was unclear whether a gland was benign or malignant, the slides that were immunohistochemically stained with p63-AMACR or 34betaE12 were retrieved from the archive, if available, and inspected. Regions in which grading was impossible due to out-of-focus, tissue folds, or excessive ink and regions in which the immunohistochemical staining was inconclusive or unavailable were excluded from the study. Moreover, all regions with high-grade prostatic intraepithelial neoplasia were excluded from the study.

### Convolutional neural network

Training a CNN requires large datasets with considerable variation, which are often not available in medical diagnostics. Here, we present a study exploiting a large amount of pixels and substantial variations in tumor glands in each WSI to train a CNN [18]. By training a CNN on detailed GP annotations, we aim to make accurate differentiation of GP and GG in heterogeneous prostate biopsies.

#### Patch generation

Patches were extracted from the annotated RGB images. The patch size was required to be 299 × 299 pixels, which correspond to an area of approximately 150 × 150 μm^2^. Patches (with possible overlap) were randomly extracted from the image using MATLAB R2015b, MathWorks, Natick MA USA. The central pixel of the patch defined the class. As CNNs require huge datasets for training, data augmentation was applied. Rotation by 90, 180, and 270°, as well as horizontal and vertical mirroring, was applied to all patches.

Based on the number of extracted patches of each class, the patches were grouped in four balanced partitions. In these balanced partitions, a biopsy could only be present in one partition. Within these partitions, the number of patches in each class was reduced to equal the smallest class in all partitions.

#### CNN architecture

The CNN was trained based on three of these balanced partitions (which added up to approximately 268,000 patches) that were designated as the training set, while the fourth partition was designated as the test set and was used for cross-validation (with approximately 89,000 patches). This procedure allowed us to study the performance of the CNN four times. The CNN (Inception v3 architecture) [22] was retrained using CNTK, which is an open source deep learning toolkit for image recognition [23]. This CNN is composed of various layers of Inception modules and two classifying layers [22]. The CNN results in a probability of a patch belonging to each of the four tissue classes. Specifications of the network can be found in Table S1.

#### Post-processing

The probabilities provided by the CNN were used to differentiate between non-atypical tissue (non-atypical gland patches with unaffected stroma patches), GP 3 and GP ≥ 4, by using a cross-validated support vector machine. Next, by assigning the probability of each patch belonging to one of these three classes, probability maps were generated. Each patch of the test set was assigned to the class according to the highest probability.

The percentages of GP 3 and GP ≥ 4 classified of randomly selected patches of a biopsy were used to classify the slides according to the adjusted GGs. Using Table 1, the majority and minority of the automatic classified GPs patches are summed up (e.g., GP ≥ 4 + GP 3 = adjusted GG 3). In case that only one GP was present, this GP was doubled (e.g., GP 3 + GP 3 = adjusted GG 1). At least 4.5% of the patches needed to be positively identified for each class (GP 3 and GP ≥ 4) to reduce the influence noise for the adjusted GG determination.

Post hoc visual evaluation of the probability maps was performed to identify possible causes of false positive regions of the methodology.

### Accuracy analysis

The three assigned classes were represented in a confusion matrix for comparison with the manually depicted class. The confusion matrix was subsequently dichotomized to calculate the sensitivity, specificity, and accuracy. The F-measure was used as an accuracy measure. The F-measure (*F*_1_) considers both precision and recall and is defined as *F*_1_ = 2 (precision × recall)/(precision + recall). The patches were dichotomized between non-atypical and malignant tissue (GP ≥ 3). Subsequently, we also assessed the accuracy for differentiating GP ≤ 3 from GP ≥ 4. This differentiation has been used as a measure to determine the need of treatment [24].

The kappa-statistic (κ) was used to calculate the concordance between the GG classifications and the reference standard. A quadratic weighted kappa was used in which disagreement on the ordinal GG scale was not assumed to be equally important [25].

## Results

Training of each CNN took approximately 175 h. Because only minor differences existed between the performance of the four trained networks, only the results of one representative trained network are illustrated. The ratio of correct-pixel-classified patches was 93% for non-atypical patches, 73% of GP 3, and 77% of GP ≥ 4. The confusion matrix of the classifications is presented in Table 2.

**Table 2:** Confusion matrix of the pixel-classified patches

		Estimated class (%)		
		Non-atypical	GP = 3	GP ≥ 4
Reference standard	Non-atypical	93	5	2
	GP = 3	14	73	13
	GP ≥ 4	7	17	77

The differentiation between non-atypical and malignant (GP ≥ 3) areas had an accuracy of 92%, with a sensitivity and specificity of 90 and 93%, respectively. The F-measure was 0.93.

The differentiation between GP ≥ 4 and GP ≤ 3 was accurate for 90%, with a sensitivity and specificity of 77 and 94%, respectively, and with an F-measure of 0.81.

**Fig. 2:**
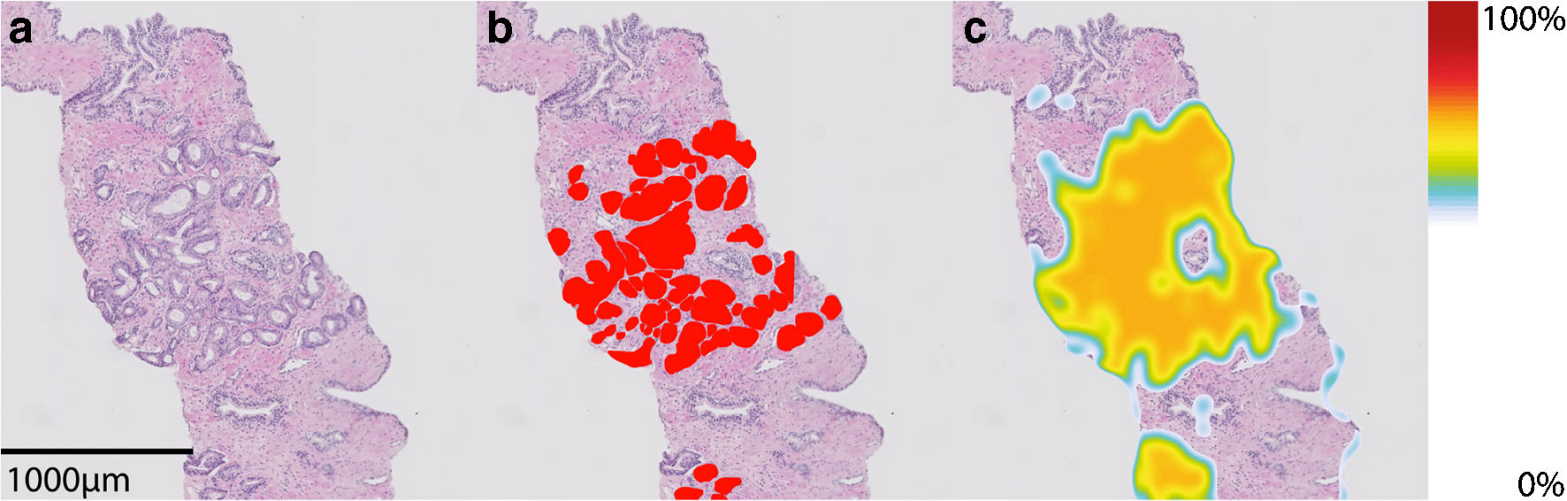
**a** H&E biopsy fragment, **b** ground truth manual annotations of GP 3, **c** probability map for malignancy. Color scale on the right of the image indicates the probability

**Fig. 3:**
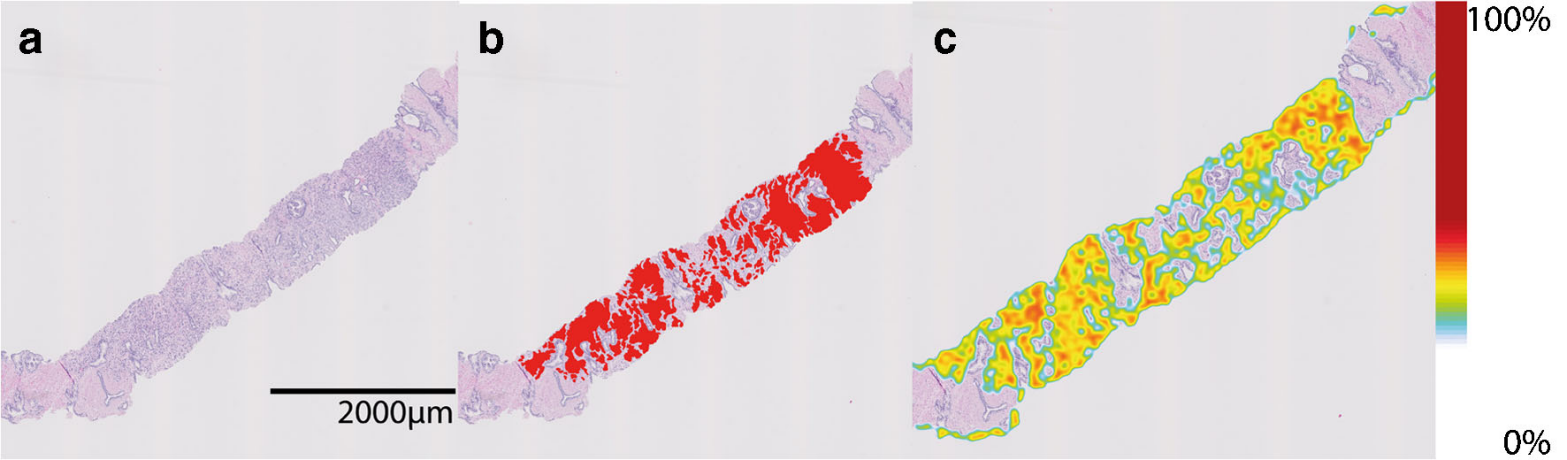
**a** H&E biopsy fragment, **b** ground truth manual annotations of GP 4, **c** probability map for GP ≥ 4. Color scale on the right of the image indicates the probability

An example of a probability map for malignant tissue is shown in Fig. 2 and a probability map for GP ≥ 4 is shown in Fig. 3. Visual inspection of the probability maps, resolved false-positive regions at tissue folds and at regions that were either out-of-focus or obscured by the presence of ink. Another major contributor to false-positive regions is the border of the biopsies. In these regions, incomplete glands as well as cutting artifacts are mostly present.

Concordance of the full biopsy-based adjusted GG classification was obtained in 65% (*N* = 40) of the biopsies, resulting in a *κ* of 0.70 (see Table 3), indicating substantial agreement.

**Table 3:** Confusion matrix of the estimated adjusted GG and the manual reference standard

		Estimated adjusted GG			
		1	2	3	4
Reference standard	1	19	4	0	1
	2	1	2	1	0
	3	2	0	2	1
	4	0	1	3	3

## Discussion

We have demonstrated that a CNN is accurate in the differentiation of GP 3 and GP ≥ 4 from non-atypical tissue for prostate biopsies with good accuracy. Probability maps of GPs showed good visual agreement, suggesting that CNNs can be a valuable tool for computer-aided diagnosis. Determination of the adjusted GG based on the presence of the GPs showed substantial agreement with the reference standard.

### Comparison with current literature

The agreement between human and automated GG classification is in line with the inter-observer agreement between two general pathologists described by Ozkan et al. [8] This conformity indicates that the GG classification agreement cannot be further improved since higher concordance with one observer would result in a lower concordance with another. Other automated methods for GP classification are mainly based on the automated detection of glands and afterwards the extraction of hand-crafted features, such as the gland and lumen surface area, for classification [12, 14, 15]. Consequently, some regions of GP 4 can be missed by these automatic detection methods, as glandular structures are largely reduced and affected here [15]. CNNs have proven to be useful for classification of prostate biopsies. Litjens et al. [18] automatically differentiated between tumorous and non-tumorous prostate biopsies. Källén et al. [20] made further efforts to differentiate between GP 3 to GP 5. In their study, they only used homogeneous single-class regions of interest and classified whole biopsies based on the most prominent GP within the slide. The patch-based performance of Källén et al. is also exceeded by our study [20]. In particular, the accuracy of the differentiation between GP 3 and GP 4 patches is higher in this study, and differentiation between GP 3 and GP 4 has the biggest clinical implications. Differentiation between GP 3 and GP 4 is very often problematic [26, 27]. For instance, fused or small glands without lumina can be categorized as either GP 3 or GP 4 [26]. The training using detailed annotations in this study might explain the improved accuracy compared with Källén et al. [20]. These detailed annotations allow accurate differentiation between the various GPs in heterogeneous biopsies and thereby mimicking the human interpretation of prostate biopsies.

### Future perspective

Automated diagnosis has the potential to reduce both the workload of and variability between pathologists [12, 13]. CNNs have already outperformed pathologists with a time-constraint in the detection of breast cancer metastasis in the lymph nodes [28].

Although the patch-based classification can be considered accurate, classification results may be improved by the introduction of extensive post-processing. By taking into account more information of the neighborhood, conditional random fields (CRFs), among others, have the potential to improve the label assignment [17].

For the generalizability of future CNNs, the dataset should be annotated by multiple genitourinary pathologists in order to decrease the influence of inter-observer variability. In this dataset, special attention should be paid to include more patients with a (heterogeneous) GP5. This would allow the system to classify according to the official GG instead of the adjusted GG. Moreover, biopsies from multiple institutions should be incorporated due to the differences in appearance of biopsies, among others by different staining protocols. The benefits of the inclusion of more biopsies from different hospitals are twofold. First of all, it makes the applied methodology more robust against differences in appearance of the biopsy, and secondly, it results in an improvement of the performance of the CNN.

### Limitations

This study suffered from a number of limitations. The differentiation between GP 3 and GP 4 was based on the annotations made by two trained observers and one expert genitourinary pathologist, although it is known from the literature that a large degree of variation may exist between the diagnoses of individual pathologists. The same holds for the annotation of LGPIN. However, we assume that the precise annotation of the glands on high-resolution images, as well as the two-staged delineation process, resulted in a reliable dataset.

As only data of 96 tissue sections from 38 different patients were included, we partitioned the data based on biopsies rather than on patients. This approach may have resulted in an overestimation of the accuracy, as patient-specific patterns can be present in both training and testing partitions. However, we found no indication of overfitting to patient-specific patterns. In patients present in only a single partition, visual inspection of these biopsies shows similar performance as patients present in multiple partitions. To improve the performance of the CNN, false-positive regions caused by tissue folds, out-of-focus, borders of the biopsy, and ink should be automatically excluded, as these regions may distract the attention from real findings. Nonetheless, the automated adjusted GG determination displayed a comparable agreement than the reported inter-observer agreement in Ozkan et al. [8], as the majority of the patients in the test-set are in GG 1. Differentiation between adjusted GG 2 and adjusted GG 3 is still challenging, while this differentiation has the largest clinical implications for patients. Unfortunately due to the low presence of GP 5, this study introduced the adjusted GG. Therefore, the proposed methodology is aimed at the localization and differentiation of GPs in whole needle biopsies. This can help the pathologists in the detection and suggest the GPs in prostate biopsies.

## Conclusions

We demonstrate the feasibility to train a CNN to differentiate between GPs in heterogeneous biopsies. Good differentiation between non-atypical tissue and tumorous tissue is achieved, as well as a substantial agreement in GG classification between the automated method and the specialized genitourinary pathologist.

## Electronic supplementary material

ESM 1 (DOCX 26.3 kb)

## References

[1] Siegel R, Miller K, Jemal A (2015). Cancer statistics , 2015. CA Cancer J Clin.

[2] Zhou CK, Check DP, Lortet-Tieulent J, Laversanne M, Jemal A, Ferlay J, Bray F, Cook MB, Devesa SS (2016). Prostate cancer incidence in 43 populations worldwide: an analysis of time trends overall and by age group. Int J Cancer.

[3] Heidenreich A, Bastian PJ, Bellmunt J, Bolla M, Joniau S, van der Kwast T, Mason M, Matveev V, Wiegel T, Zattoni F, Mottet N (2014). EAU guidelines on prostate cancer. Part 1: screening, diagnosis, and local treatment with curative intent - update 2013. Eur Urol.

[4] Epstein JI, Allsbrook WCJ, Amin MB, Egevad LL (2005). The 2005 International Society of Urological Pathology (ISUP) consensus conference on Gleason grading of prostatic carcinoma. Am J Surg Pathol.

[5] Epstein JI, Zelefsky MJ, Sjoberg DD, Nelson JB, Egevad L, Magi-Galluzzi C, Vickers AJ, Parwani AV, Reuter VE, Fine SW, Eastham JA, Wiklund P, Han M, Reddy CA, Ciezki JP, Nyberg T, Klein EA (2016). A contemporary prostate Cancer grading system: a validated alternative to the Gleason score. Eur Urol.

[6] Samaratunga H, Delahunt B, Gianduzzo T, Coughlin G, Duffy D, LeFevre I, Johannsen S, Egevad L, Yaxley J (2015). The prognostic significance of the 2014 International Society of Urological Pathology (ISUP) grading system for prostate cancer. Pathology.

[7] Epstein JI (2017). Prostate Cancer grade groups correlate with prostate-specific Cancer mortality: SEER data for contemporary graded specimens. Eur Urol.

[8] Ozkan TA, Eruyar AT, Cebeci OO, Memik O, Ozcan L, Kuskonmaz I (2016). Interobserver variability in Gleason histological grading of prostate cancer. Scand J Urol.

[9] Nakai Y, Tanaka N, Shimada K, Konishi N, Miyake M, Anai S, Fujimoto K (2015). Review by urological pathologists improves the accuracy of Gleason grading by general pathologists. BMC Urol.

[10] Egevad L, Ahmad AS, Algaba F, Berney DM, Boccon-Gibod L, Compérat E, Evans AJ, Griffiths D, Grobholz R, Kristiansen G, Langner C, Lopez-Beltran A, Montironi R, Moss S, Oliveira P, Vainer B, Varma M, Camparo P (2013). Standardization of Gleason grading among 337 European pathologists. Histopathology.

[11] Evans SM, Patabendi Bandarage V, Kronborg C, Earnest A, Millar J, Clouston D (2016). Gleason group concordance between biopsy and radical prostatectomy specimens: a cohort study from prostate Cancer outcome registry ? Victoria. Prostate Int.

[12] Doyle S, Hwang M, Shah K et al (2007) Automated grading of prostate cancer using architectural and textural image features. 2007 4th IEEE Int Symp Biomed Imaging From Nano to Macro - Proc:1284–1287. 10.1109/ISBI.2007.357094

[13] Vennalaganti PR, Naag Kanakadandi V, Gross SA (2015). Inter-observer agreement among pathologists using wide-area Transepithelial sampling with computer-assisted analysis in patients with Barrett’s esophagus. Am J Gastroenterol.

[14] Nguyen K, Jain AK, Allen RL (2010) Automated gland segmentation and classification for Gleason grading of prostate tissue images. 2010 20th Int Conf pattern Recognit:1497–1500. 10.1109/ICPR.2010.370

[15] Naik S, Doyle S, Feldman M et al (2007) Gland segmentation and computerized Gleason grading of prostate histology by integrating low- , high-level and domain specific information. Proc 2nd Work Microsopic Image Anal with Appl Biol:1–8

[16] Montironi R, Cheng L, Lopez-Beltran A, Mazzucchelli R, Scarpelli M, Bartels PH (2009). Decision support systems for morphology-based diagnosis and prognosis of prostate neoplasms: a methodological approach. Cancer.

[17] Litjens G, Kooi T, Bejnordi BE et al (2017) A Survey on Deep Learning in Medical Image Analysis. 10.1016/j.media.2017.07.005 10.1016/j.media.2017.07.005 28778026

[18] Litjens G, Sánchez CI, Timofeeva N, Hermsen M, Nagtegaal I, Kovacs I, Hulsbergen - van de Kaa C, Bult P, van Ginneken B, van der Laak J (2016) Deep learning as a tool for increased accuracy and efficiency of histopathological diagnosis. Sci Rep 6(26286). 10.1038/srep26286 10.1038/srep26286 PMC4876324 27212078

[19] Ing N, Ma Z, Li J et al (2018) Semantic segmentation for prostate cancer grading by convolutional neural networks. Med Imaging 2018 Digit Pathol:46. 10.1117/12.2293000

[20] Källén H, Molin J, Heyden A et al (2016) Towards grading Gleason score using generically trained deep convolutional neural networks. In: 2016 IEEE 13th international symposium on biomedical imaging (ISBI), IEEE, pp 1163–1167

[21] Kamphuis G, de Bruin D, Brandt M et al (2016) Comparing Image Perception of Bladder Tumours in Four Different Storz Professional Image Enhancement System ( SPIES ) Modalities using the íSPIES App. 30:1–20. 10.1089/end.2015.0687 10.1089/end.2015.0687 26743929

[22] Szegedy C, Vanhoucke V, Ioffe S et al (2015) Rethinking the inception architecture for computer vision. 10.1109/CVPR.2016.308

[23] Yu D, Eversole A, Seltzer M et al (2014) An introduction to computational networks and the computational network toolkit. 112

[24] Poel Van Der HG (2016). Difference of opinion active surveillance in intermediate risk prostate cancer : is it safe ? Opinion : yes. BJU Int.

[25] Vanbelle S (2016). A new interpretation of the weighted kappa coefficients. Psychometrika.

[26] Kweldam CF, Nieboer D, Algaba F, Amin MB, Berney DM, Billis A, Bostwick DG, Bubendorf L, Cheng L, Compérat E, Delahunt B, Egevad L, Evans AJ, Hansel DE, Humphrey PA, Kristiansen G, van der Kwast TH, Magi-Galluzzi C, Montironi R, Netto GJ, Samaratunga H, Srigley JR, Tan PH, Varma M, Zhou M, van Leenders GJLH (2016). Gleason grade 4 prostate adenocarcinoma patterns: an interobserver agreement study among genitourinary pathologists. Histopathology.

[27] Latour M, Amin MB, Billis A, Egevad L, Grignon DJ, Humphrey PA, Reuter VE, Sakr WA, Srigley JR, Wheeler TM, Yang XJ, Epstein JI (2008). Grading of invasive cribriform carcinoma on prostate needle biopsy: an interobserver study among experts in genitourinary pathology. Editorial comment Am J Surg Pathol.

[28] Bejnordi BE, Veta M, Van Diest PJ (2017). Diagnostic assessment of deep learning algorithms for detection of lymph node metastases in women with breast cancer. JAMA - J Am Med Assoc.

