# Supplementary material for: Deep learning for automatic Gleason pattern classification for grade group determination of prostate biopsies
Source: Virchows Arch. 2019 May 16;475(1):77–83. doi: 10.1007/s00428-019-02577-x (PMC6611751; doi:10.1007/s00428-019-02577-x)
Supplement: Supplementary file 1 — (DOCX 26.3 kb) [file 428_2019_2577_MOESM1_ESM.docx]

**Supporting information**

Table S1: Specification on the network in the training phase.

| **Specification of the network** | |
| --- | --- |
| **Dropout rate** | 0.40 |
| **Nr of epochs** | 600 |
| **Nr of patches/epoch** | 40,000 |
| **Minibatch size** | 25 |
| **Momentum** | 0.9 |
